# Circular RNA as biomarkers for acute ischemic stroke: A systematic review and meta‐analysis

**DOI:** 10.1111/cns.14220

**Published:** 2023-04-26

**Authors:** Xiao Zhang, Mengyao Wan, Xiaoli Min, Guanglei Chu, Yumin Luo, Ziping Han, Wei Li, Ran Xu, Jichang Luo, Wenjing Li, Yutong Yang, Yan Ma, Liqun Jiao, Tao Wang

**Affiliations:** ^1^ Department of Neurosurgery, Xuanwu Hospital Capital Medical University Beijing China; ^2^ Sir William Dunn School of Pathology University of Oxford Oxford UK; ^3^ China International Neuroscience Institute (China‐INI) Beijing China; ^4^ Peking Union Medical College Chinese Academy of Medical Sciences Beijing China; ^5^ Department of Cerebrovascular Diseases, The Second Affiliated Hospital Kunming Medical University Kunming China; ^6^ Department of Neurology The Second Affiliated Hospital of Zhengzhou University Zhengzhou China; ^7^ Institute of Cerebrovascular Diseases Research and Department of Neurology, Xuanwu Hospital Capital Medical University Beijing China; ^8^ Department of Neurosurgery Liaocheng People's Hospital Liaocheng China; ^9^ National Laboratory of Pattern Recognition, Institute of Automation Chinese Academy of Sciences Beijing China; ^10^ Department of Interventional Neuroradiology, Xuanwu Hospital Capital Medical University Beijing China

**Keywords:** acute ischemic stroke, biomarkers, circular RNA

## Abstract

**Background:**

Rapid diagnosis of acute ischemic stroke (AIS) patients is still challenging, and reliable biomarkers are needed. Noncoding RNAs are important for many physiological activities, among which circular RNAs (circRNAs) have been proven to be more tissue‐specific and conservative. Many recent studies found the potential of circRNAs as biomarkers for many diseases, including cardiovascular diseases, cancers, and ischemic stroke. This systemic review and meta‐analysis aimed to identify circRNAs as potential biomarkers for AIS.

**Methods:**

This study has been prospectively registered in PROSPERO (Registration No. 11 CRD42021288033). Published literature comparing circRNA expression profiles between AIS and non‐AIS in human and animal models were retrieved from the articles published by January 2023 in major databases. We descriptively summarized the included studies, conducted meta‐analysis under a random effects model, and did bioinformatics analysis including Gene Ontology (GO) and Kyoto Encyclopedia of Genes and Genomes (KEGG) enrichment analysis.

**Results:**

Totally 23 studies were included, reporting 18 distinctive upregulated and 20 distinctive downregulated circRNAs. Diagnostic meta‐analysis indicated discriminative ability of the circRNAs. Furthermore, circRNA *HECTD1*, circRNA *DLGAP4*, circRNA *CDC14A*, circRNA *SCMH1*, and circRNA *TLK1* were reported with the same regulation trend in more than one study (animal studies included). GO and KEGG enrichment analyses indicated that the target genes of these five circRNAs were enriched in regulating cell proliferation, apoptosis, and oxidative stress.

**Conclusions:**

This study demonstrates that circRNAs (circRNA *HECTD1*, circRNA *DLGAP4*, circRNA *CDC14A*, circRNA *SCMH1*, and circRNA *TLK1*) generally are promising as potential biomarkers for AIS. However, due to the limited number of studies, diagnostic value of individual circRNA could not be validated. More in vitro and in vivo functional studies are needed.

## Graphical abstract

This review updated the latest knowledge about the role of circular RNAs in the development of acute ischemic stroke, and discussed the practicability of using them as the biomarker for diagnosis. This study will pave the way for future studies of competing endogenous RNAs in ischemic stroke and furthermore, comprehension of the molecular mechanisms behind stroke.

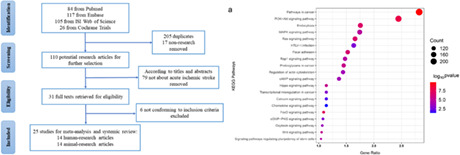

## 1 INTRODUCTION

The incidence of cerebrovascular diseases is increasing worldwide, of which acute ischemic stroke (AIS) has high mortality and morbidity, making it one of the top causes of death.^1^ The early and accurate diagnosis of AIS and early beginning of treatment greatly improve AIS patients' prognosis. And understanding the molecular mechanisms behind AIS could help to identify applicable diagnostic biomarkers.

Increasing evidence has proved that noncoding RNAs play important roles in many physiological and pathophysiological processes.^2^, ^3^ The circular RNA (circRNA) is a subclass of noncoding RNAs, characterized by a covalently closed loop that enables it to escape the degradation by nuclease.^4^ Furthermore, circRNAs were found to be more tissue‐specific and evolutionally conserved. Current studies demonstrated that circRNAs functioned through combining with miRNAs or proteins and reacting with RNA polymerase II, thus regulating epigenetic modification and transcription.^3^, ^5^ Besides, some circRNAs could encode proteins with an insertion of internal ribosome entry site in the upstream of start codons.^6^ Research and meta‐analyses found that certain kinds of circRNAs were associated with cancer,^7^, ^8^ cardiovascular diseases,^9^ and other diseases, which indicated its possible utility in disease diagnosis and treatment. In recent years, several studies explored the differences in circRNA profiles between AIS patients and healthy people. However, there is inconsistency among these studies, concerning research technologies, statistical analysis methods, cut‐off value, and so on. Therefore, it could be challenging to integrate individual results and comprehend the function of circRNAs in AIS. Systemic review and meta‐analysis could be effective and optimal approaches to combine results from various studies. Therefore, we did this study on the published and registered research that explored the differently expressed circRNAs in AIS patients and animal models of AIS, hoping to provide information for future clinical application of circRNAs.

## 2 METHODS

The present study has been prospectively registered in PROSPERO (Registration No. 11 CRD42021288033) and was developed following the Preferred Reporting Items for Systematic Review and Meta‐Analysis Protocols statement (PRISMA checklist and abstract checklist).

### 2.1 Search strategies

Four major English databases, PubMed, EMBASE, ISI web of science, and Cochrane Library were thoroughly searched to investigate the association between circRNA profiling and AIS. The following terms were used: “circular RNA” or “circRNA,” and “stroke” or “cerebral infarction” or “cerebral ischemia” or “cerebral vascular accident” or “brain infarction” or “brain ischemia” or “brain vascular accident” from January 2010 to January 2023. Two authors carefully examined the titles and abstracts of retrieved records, and any disagreements were resolved by consensus.

### 2.2 Selection standards

Eligible studies had to meet the following inclusion criteria: (a) circRNA expression studies on AIS patients with approximate age‐ and sex‐matching healthy controls for comparison; (b) circRNA expression studies on rodents of stroke model with age‐ and sex‐matching healthy mice/rats for comparison; (c) using real‐time quantitative reverse transcription polymerase chain reaction (qRT‐PCR), circRNA microarray, and/or RNA‐sequencing technologies; and (d) reporting selection criteria of differentially expressed circRNAs.

For participant inclusion, patients must: (a) be confirmed of AIS with magnetic resonance imaging or computer tomography of the head; (b) have biological specimens acquired within 72 h after AIS onset and before any treatment. Patients and healthy controls with the following situations were excluded: (a) concomitant with other diseases, including acute infections, immunological diseases, neurodegenerative diseases, and cancer, (b) taking prior medication with low molecular weight or unfractionated heparin within the last month, (c) cerebral hemorrhage secondary to stroke.

For animal experiments, animals used should: (a) be adult, and have detailed reports of feeding condition; (b) have no gene‐edition, medication, or any other treatment; (c) have histological and/or behavioral confirmation of brain damage after occlusion; (d) have specimens acquired within 24 hours after occlusion.

### 2.3 Data extraction and quality assessment

Information from the full texts and supplementary files of the selected studies were collected, which included: first author, publication year, selection of AIS patients and healthy controls, research technologies, cut‐off criteria and fold changes (FC), names, and analysis of the significantly dysregulated circRNAs and so forth. When values were not provided in articles or supplementary materials, the extraction of statistical data from graphs was performed with WebPlotDigitizer (Version 4.4). Besides, the accuracy of this method was verified with studies providing both graphs and detailed values.

Since there is no unified nomination of circRNAs, we retained the names of circRNA in the original studies, and meanwhile searched for unified nomination on circBASE (http://circrna.org/). UCSC (genome.ucsc.edu/) and CIRCexplorer2 (www.picb.ac.cn/rnomics/circpedia/) were referred for the corresponding gene and positions of the circRNAs.

The cut‐off threshold was set as |FC| > 1.5 or <0.6, and *p* < 0.05. Because there is no scale to evaluate all the used platforms, the new quality assessment scale from Li′s meta‐analysis^9^ was adopted, which consisted of five parts: (a) course design, (b) detailed description of the samples, (c) description and representation of case and control, (d) annotation of the platform and naming convention of circRNAs, and (e) raw data processing and data analysis. Full score for each part was 2, 0 for undescribed part, while 1 for incomplete description. Furthermore, Quality Assessment of Diagnostic Accuracy Studies 2 (QUADAS‐2)^10^ was adopted to evaluate the included studies for the diagnosis value of circRNAs. The QUADAS‐2 consists of four main domains, patient selection, index test, reference standard, and flow and timing, each of which is assessed in terms of risk of bias as “low,” “high,” or “unclear.” The assessment was done by two qualified members of our team, and consistency was reached for final score.

### 2.4 Statistical analysis

Data collection and organization were done with Excel (2019 Microsoft). Statistical analysis of the diagnostic tests was executed STATA 15.1. *Q*‐tests and *I* ^2^ statistics were used to estimate the heterogeneity with either *p* < 0.10 or *I* ^2^ > 50% suggesting the existence of substantial heterogeneity. A random‐effects model was applied to quantify the pooled sensitivity, specificity, log diagnostic odds ratio (LDOR), and area under curve (AUC), with corresponding 95% confidence intervals (CIs). Spearman correlation analysis was conducted to verify the threshold effects. For the test of robustness, a sensitivity analysis was performed to attain more accurate results. Publication bias was evaluated with Deek's funnel plots. All tests were two‐tailed and *p* < 0.05 was considered statistically significant.

### 2.5 Analysis for secondary outcome

The circRNAs reported in several studies were further selected for bioinformatic analysis. According to ceRNA theory, the targeted miRNAs for circRNAs were predicted with CircInteractome database, and the targeted mRNA/genes were predicted with miRDB database. The competing endogenous RNAs (ceRNA) network for the first‐ranking miRNA and the top 20 mRNAs were demonstrated with Cytoscape (version 3.9.0). Furthermore, we used Gene Ontology (GO) and Kyoto Encyclopedia of Genes and Genomes (KEGG) to predict the functions of the top‐ranking targeted genes. GO (geneontology.org/) and KEGG (kegg.jp/) are public online database, the former providing information of biological process (BP), cellular components (CC), and molecular functions (MF) of the target genes, while the later analyzing the pathways involved.

## 3 RESULTS

### 3.1 Characteristics of included studies

**FIGURE 1:**
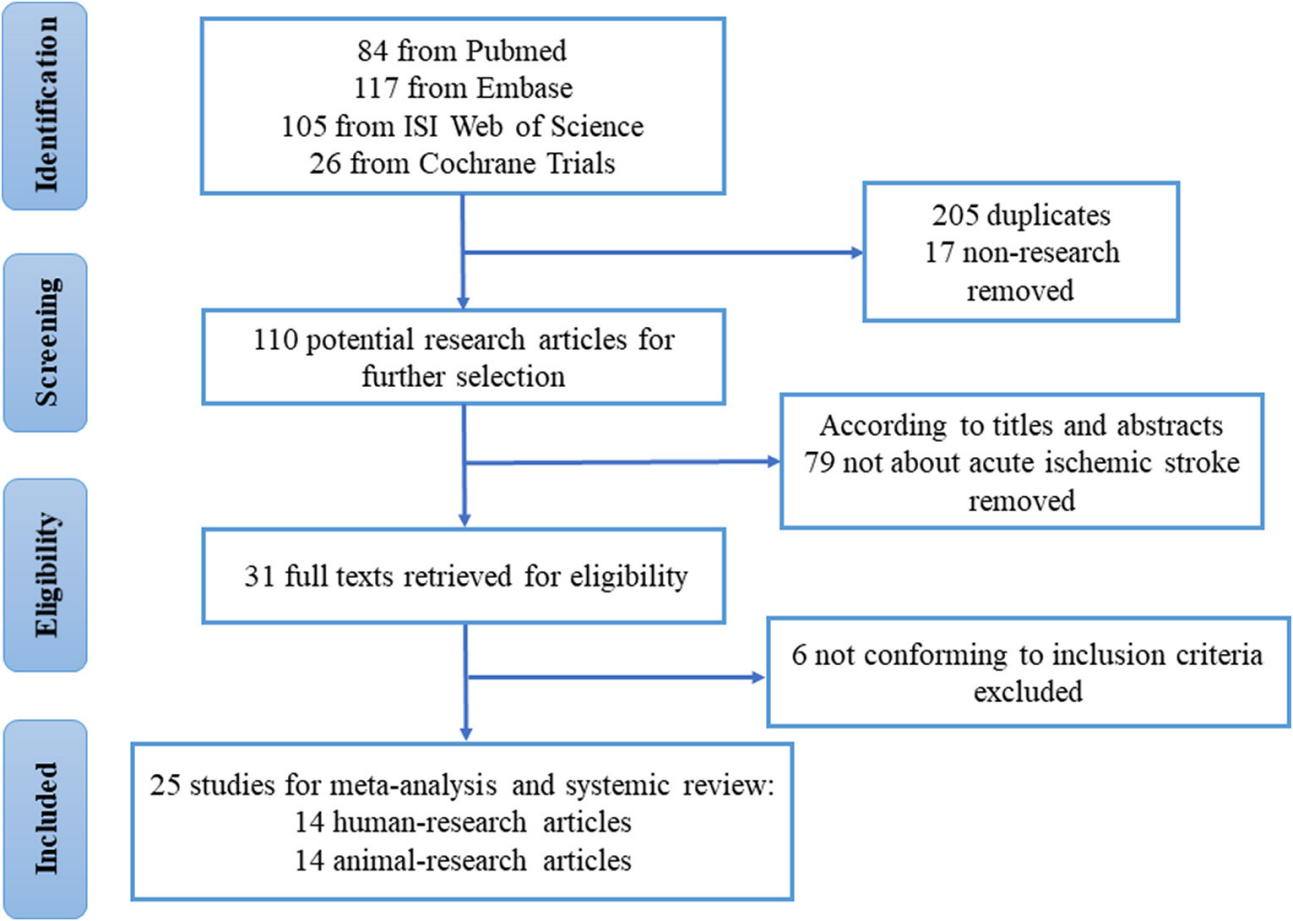
Flow diagram of literature inclusion. Identification, screening, eligibility extraction, and inclusion steps of studies were depicted.

Figure 1 illustrated the flow diagram of literature search and study selection. In total, 23 studies were included, among which, nine explored the circRNA expression profiles of human only and nine of animals. Five studies verified the differentially expressed circRNAs on both human and animal samples. Table 1 showed the information of the included studies, and the quality evaluation of the included studies was shown in Tables S1 and S2. Most studies lacked or were insufficient in describing the prespecified thresholds, standard reference tests and annotating the circRNAs. Among these studies, 10 had divided experiments into exploration section, with microarray or RNA sequencing, and verification section, with qRT‐PCR. These studies chose random or the top ranking circRNAs differentially expressed in sequencing data for further PCR verification.

**TABLE 1:** General information of the included studies.

Article	Species	Specimen	AIS			Control			Tech	DE circRNA	
			*N*	Age	Sex (M)	*N*	Age	Sex (M)		Up	Down
XP Peng (2019)^15^	Human	PBMCs	160	62.5 ± 12.9	109	160	60.6 ± 10.7	114	qRT‐PCR	1	/
XQ Zhu (2019)^32^	Human	PBMCs	170	64.9 ± 12.5	124	170	63.9 ± 10.5	131	qRT‐PCR	/	1
Y Chen (2020)^34^	Human	Serum	80	57.0 ± 21.1	53	30	56.0 ± 20.9	20	Microarray	4	4
									qRT‐PCR	1	/
ZF Dong (2020)^35^	Human	PBMCs	5	47–69	4	5	45–62	3	Sequencing	373	148
									qRT‐PCR	4	4
SH Li (2020)^36^	Human	Whole blood	3	NA	NA	3	NA	NA	Sequencing	659	1611
			32	NA	NA	32	NA	NA	qRT‐PCR	1	2
Y Zhao (2020)^37^	Human	Serum	90	NA	NA	75	NA	NA	qRT‐PCR	/	1
L Zuo (2020)^19^	Human	Whole blood	3	63.0 ± 1.5	1	3	59.3 ± 2.6	1	Microarray	10	68
			36	66.3 ± 13.3	17	36	66.4 ± 13.2	17	qRT‐PCR	3	/
			200	61–82	NA	100	59–71	NA	qRT‐PCR	3	/
SN Li (2021)^38^	Human	PBMCs	5	60.33 ± 9.53	3	4	58.55 ± 8.55	2	Sequencing	182	176
			30	62.16 ± 8.95	16	30	61.25 ± 9.07	15	qRT‐PCR	3	3
			168	62.53 ± 8.70	87	118	61.84 ± 8.93	65	qRT‐PCR	1	/
Q Xiao (2021)^33^	Human	Plasma (exosomes)	5	NA	NA	5	NA	NA	Sequencing	9	16
			32	NA	NA	32	NA	NA	qRT‐PCR	/	5
L Yang (2020)^20^	Human	Plasma	3	NA	NA	3	NA	NA	Microarray	4	16
			12	NA	NA	12	NA	NA	qRT‐PCR	5	4
			145	60–75	80	145	61–82	84	qRT‐PCR	/	1
	C57BL/6J mice	Plasma	6	8–10 w	All	6	8–10 w	All	qRT‐PCR	/	1
Y Bai (2018)^39^	Human	Plasma	26	65.4 ± 2.03	13	26	66.9 ± 2.30	13	qRT‐PCR	/	1
	C57BL/6J mice	Ipsilateral hemisphere	NA	8–10 w	All	NA	8–10 w	All	qRT‐PCR	/	1
B Han (2018)^14^	Human	Plasma	37	68.50 ± 1.36	18	34	70.19 ± 1.42	19	qRT‐PCR	1	/
	C57BL/6J mice	Ischemic cortex	3	6–8 w	All	3	6–8 w	All	qRT‐PCR	1	/
FF Wu (2019)^21^	Human	Plasma	71	68.56 ± 1.07	59	71	67.86 ± 1.30	59	qRT‐PCR	1	/
	C57BL/6J mice	Plasma & ischemic cortex	13&8	8–10 w	All	13&8	8–10 w	All	qRT‐PCR	1	/
D Lu (2020)^40^	Human	Whole blood	8	55.4 ± 12.3	7	8	54.11 ± 10.1	7	qRT‐PCR	/	2
	Balb/c mice	Whole blood	3	10–12 w	All	3	10–12 w	All	Microarray	Totally 198	
		Blood & ischemic brain	3	10–12 w	All	3	10–12 w	All	qRT‐PCR	3	2
SL Mehta (2017)^41^	C57BL/6 mice	Ischemic penumbral cortex	3	12 w	All	3	12 w	All	Microarray	8	8
									qRT‐PCR	3	3
XL Yang (2018)^42^	C57BL/6 mice	Brain cortical tissues	10	8–10 w	All	10	8–10 w	All	qRT‐PCR	1	/
WH Chen (2020)^27^	C57BL/6 mice	Brain tissues	6	6–8 w	All	6	6–8 w	All	qRT‐PCR	/	1
CG Tang (2020)^43^	C57BL/6 mice	Hippocampus	NA	6–8 w	All	NA	6–8 w	All	qRT‐PCR	1	/
LQ Wu (2020)^44^	C57BL/6 mice	Brain tissues	5	8–10 w	All	5	8–10 w	All	qRT‐PCR	/	1
ZH Zhang (2020)^45^	Sprague Dawley rats	Neocortex of left frontal pole	4	NA	All	4	NA	All	Sequencing	16	28
									qRT‐PCR	3	5
QD Dai (2021)^13^	C57BL/6 mice	Brain tissues & serum	3	2 m	All	3	2 m	All	qRT‐PCR	1	/
B Yang (2021)^46^	C57BL/6 mice	Brain tissues	5	6 w	All	5	6 w	All	qRT‐PCR	1	/
ZD Zhang (2021)^47^	Vital River Lab mice	Brain tissues	NA	10 w	All	NA	10 w	All	qRT‐PCR	1	/

### 3.2 Summary of differentially expressed circRNAs

Table 2 showed details into circRNAs verified with PCR of the included studies, including FC, *P*, and the originating genes and positions of the circRNAs. In total, 18 distinctive upregulated and 20 distinctive downregulated circRNAs were reported in human studies, among which the upregulated ones, circ *HECTD1* and circ *CDC14A*, and the downregulated one, circ *DLGAP4*, were reported in more than one study. These circRNAs spread across the autosomes, and two were in X chromosome, hsa_circ_0007290 in *FUNDC1* gene and hsa_circ_0090002 in *PHKA2* gene. Since circRNAs were proven to be highly conserved in different species, animal experiments could be illustrative. Twelve distinctive upregulated and 14 distinctive downregulated circRNAs were reported in animal studies. Circ *HECTD1*, circ *RBM33*, circ *DLGAP4*, circRNA *TLK1*, and circ *SCMH1* were shown to be differentially expressed both in human and rodent. However, circ *RBM33* were reported to be upregulated in AIS patients' plasma, but downregulated in the blood and brain tissue of mice. While circRNA *CDC14A* was reported in two independent human studies.

**TABLE 2:** Details of the circular RNAs reported in the included studies.

Study	Time relapse of enrollment (occlusion & reperfusion)	Circular RNA	FC	*p*	AUC (95% CI)	Cut‐off	Sensitivity	Specificity	Regulation
XP Peng (2019)^15^	Within 24 h	circRNA *HECTD1* (chr14)	2.202	<0.001	0.814 (0.768, 0.859)	1.238	0.725	0.725	Up
XQ Zhu (2019)^32^	Within 24 h	circRNA *DLGAP4* (chr20)	0.366^a^	<0.001	0.816 (0.772, 0.859)	/^c^	0.812	0.671	Down
Y Chen (2020)^34^	Within 48 h	hsa_circ_0141720 (chr15: intergenic)	9.083^a^	<0.001	0.911 (0.892, 0.982)	2.03	0.897	0.956	Up
ZF Dong (2020)^35^	24–72 h	circRNA *TMEM56* (hsa_circ:chr1:95609447–95616975)	3.222^a^	<0.05	/^c^	/^c^	/^c^	/^c^	Up
		circRNA *TREM1* (hsa_circ:chr6:41250133–41250489)	4.444^a^	<0.05	/^c^	/^c^	/^c^	/^c^	Up
		circRNA *PLXNC1* (hsa_circ:chr12:94613792–94621011)	3.357^a^	<0.05	/^c^	/^c^	/^c^	/^c^	Up
		circRNA *PIGB* (hsa_circ: chr15:55640530–55640923)	1.935^a^	<0.05	/^c^	/^c^	/^c^	/^c^	Up
		circRNA *CEP78* (hsa_circ:chr9:80869752–80879232)	0.219^a^	<0.05	/^c^	/^c^	/^c^	/^c^	Down
		circRNA *DAB1* (hsa_circ:chr1:58971728–59004978)	0.429^a^	<0.05	/^c^	/^c^	/^c^	/^c^	Down
		circRNA *KLRK1* (hsa_circ:chr12:10539502–10546466)	0.392^a^	<0.05	/^c^	/^c^	/^c^	/^c^	Down
		circRNA *RAP1B* (hsa_circ:chr12:69044180–69048032)	0.211^a^	<0.05	/^c^	/^c^	/^c^	/^c^	Down
SH Li (2020)^36^	NA	hsa_circ_0005548 (chr11: *AMBRA1*)	2.357	0.04	0.51 (0.36, 0.66)	61.590^b^	0.594^b^	0.500^b^	Up
		hsa_circ_0000607 (chr15: *VPS13C*)	0.496	0.005	0.75 (0.63, 0.87)	5.566^b^	0.781^b^	0.625^b^	Down
		hsa_circ_0002465 (chr6: *CD109*)	0.520	0.02	0.69 (0.56, 0.82)	4.768^b^	0.750^b^	0.531^b^	Down
Y Zhao (2020)^37^	Within 24 h	hsa_circ_0072309 (chr5: *LIFR*)	0.422^a^	<0.0001	0.9505 (0.9187, 09823)	0.329	0.9823	0.9187	Down
L Zuo (2020)^19^	Within 72 h	hsa_circ_0007290 (chrX: *FUNDC1*) (*n* = 200/100)	3.67	0.00015	/^c^	/^c^	/^c^	/^c^	Up
			2.40^a^	0.00014	0.796	/^c^	/^c^	/^c^	
		hsa_circ_0004494 (chr13: *PDS5B*) (*n* = 200/100)	5.01	10^−6^	/^c^	/^c^	/^c^	/^c^	Up
			3.25^a^	4.1 × 10^−9^	0.841	/^c^	/^c^	/^c^	
		hsa_circ_0000097 (chr1: *CDC14A*) (*n* = 200/100)	6.30	3.9 × 10^−7^	/^c^	/^c^	/^c^	/^c^	Up
			3.50^a^	1.9 × 10^−9^	0.872	/^c^	/^c^	/^c^	
		3 circRNAs above combined			0.875	/^c^	0.715	0.91	
SN Li (2021)^38^	96 (75, 118) min	hsa_circ_0001460 (chr4: *NEIL3*)	2.789^a^	<0.001	/^c^	/^c^	/^c^	/^c^	Up
		hsa_circ_0001599 (*n* = 30) (chr6: *FKBP5*) (*n* = 168/118)	3.500^a^	<0.001	/^c^	/^c^	/^c^	/^c^	Up
			3.381^a^	<0.001	0.805 (0.748, 0.862)	3.42	0.6441	0.8983	
		hsa_circ_0004338 (chr9: *CAMSAP11*)	1.664^a^	<0.05	/^c^	/^c^	/^c^	/^c^	Up
		hsa_circ_0007637 (chr16: *CREBBP*)	0.482^a^	<0.001	/^c^	/^c^	/^c^	/^c^	Down
		hsa_circ_0006911 (chr2: *PPP1R21*)	0.545^a^	<0.05	/^c^	/^c^	/^c^	/^c^	Down
Q Xiao (2021)^33^	Within 72 h	hsa_circ_0000698 (chr16: *PHKB*)	0.246^a^	0.033	/^c^	/^c^	/^c^	/^c^	Down
		hsa_circ_0005585 (chr5: *NNT*)	0.362^a^	0.037	0.732	/^c^	0.773^a^	0.570^a^	Down
		hsa_circ_0043837 (chr17: *ATP6V0A1*)	0.376^a^	0.035	/^c^	/^c^	/^c^	/^c^	Down
		4 circRNAs above combined			0.862	/^c^	0.874^a^	0.786^a^	
		hsa_circ_0010155 (chr16: *NBPF1*)	0.574^a^	0.026	0.822	/^c^	0.756^a^	0.786^a^	Down
L Yang (2020)^20^	Withing 72 h	circRNA *CDC14A* (chr1)	5.667^a^	<0.05	/^c^	/^c^	/^c^	/^c^	Up
		circRNA *CCZ1* (chr7)	6.207^a^	<0.01	/^c^	/^c^	/^c^	/^c^	Up
		circRNA *RBM33* (chr7)	5.200^a^	<0.01	/^c^	/^c^	/^c^	/^c^	Up
		circRNA *ZCCHC11* (chr1)	4.769^a^	<0.01	/^c^	/^c^	/^c^	/^c^	Up
		circRNA *CDC42BPA* (chr1)	3.769^a^	<0.01	/^c^	/^c^	/^c^	/^c^	Up
		circRNA *GNB2L1* (chr5)	0.400^a^	<0.05	**/**	/^c^	/^c^	/^c^	Down
		circRNA *FBXW4* (chr10)	0.316^a^	<0.05	/^c^	**/**	/^c^	/^c^	Down
		circRNA *PDE4B* (chr1)	0.370^a^	<0.05	/^c^	/^c^	/^c^	/^c^	Down
		circRNA *SCMH1* (*n* = 12)	0.327^a^	<0.05	/^c^	/^c^	/^c^	/^c^	Down
		(chr:1) (*n* = 145)	0.600^a^	<0.001	0.7896	/^c^	0.9172	0.6483	
	12 h after occlusion	circRNA *SCMH1*	0.562^a^	<0.05					Down
Y Bai (2018)^39^	16.5 ± 2.6 h	circRNA *DLGAP4* (chr20)	0.6	<0.001	/^c^	/^c^	/^c^	/^c^	Down
	24 h after occlusion	mm9_circ_015028 (chr2: *DLGAP4*)	0.325^a^	0.004					Down
B Han (2018)^14^	16.5 ± 2.6 h	circRNA *HECTD1* (chr14)	1.58^a^	<0.001	/^c^	/^c^	/^c^	/^c^	Up
	12 h after occlusion	mm9_circ_008488 (chr12: *HECTD1*)	1.58^a^	<0.05					Up
FF Wu (2019)^21^	Within 24 h	circRNA *TLK1* (chr2)	5.067	<0.0001	0.868	2.2065	0.789	0.915	Up
	6 h after occlusion	mm9_circ_009932 (plasma) (chr2: *TLK1*) (ischemic cortex)	2.664	0.0136					Up
			4.013	0.0013					
D Lu (2020)^40^	179 (60, 280) min	hsa_circ_0090002 (chrX: *PHKA2*)	8.53^b^	0.001	/^c^	/^c^	/^c^	/^c^	Down
		hsa_circ_0039457 (chr16: *BBS2*)	2.08^b^	0.01	/^c^	/^c^	/^c^	/^c^	Down
	NA	mmu_ circ_45876 (chrX: *CNKSR2*)	4.31^b^	<0.05					Up
		mmu_circ_24344 (chr11: *PSMD12*)	1.91^b^	<0.05					Up
		mmu_circ_38328 (chr5: *RBM33*)	0.42^b^	<0.01					Down
		mmu_circ_26316 (chr13)	0.51^b^	<0.01					Down
SL Mehta (2017)^41^	6 h after occlusion	mmu_circ_008018 (chr4: *PUM1*)	10.41^a^	<0.05					Up
		mmu_circ_015350 (chr1: *NCOA2*)	2.38^a^	<0.05					Up
		mmu_circ_016128 (chr7: *NARS2*)	6.25^a^	<0.05					Up
		mmu_circ_011137 (chr14: *R3HCC1*)	0.454^a^	<0.05					Down
		mmu_circ_001729 (chr9: *RASA2*)	0.336^a^	<0.05					Down
		mmu_circ_006696 (chr12: *STRN3*)	0.484^a^	<0.05					Down
XL Yang (2018)^42^	24 h after occlusion	mmu_circ_008018 (chr19: *FLRT1*)	2.752^a^	<0.05					Up
WH Chen (2020)^27^	NA	circRNA *UCK2* (chr1)	0.398^a^	<0.05					Down
CG Tang (2020)^43^	24 h after occlusion	mmu_circ_016719 (chrX: *HUWE1*)	3.610^a^	<0.001					Up
LQ Wu (2020)^44^	24 h after occlusion	circRNA *CCDC9* (chr7)	0.225^a^	<0.05					Down
ZH Zhang (2020)^45^	3 h after occlusion	circRNA *CAMK4* (chr18)	1.647^a^	<0.01					Up
		circRNA *MCTP1* (chr13)	2.651^a^	<0.01					Up
		circRNA *EPHB1* (chr9)	0.025^a^	<0.001					Down
		circRNA *GLI3* (chr13)	0.140^a^	<0.001					Down
		circRNA *RGD1310951*	0.249^a^	<0.001					Down
		circRNA *GUCY1A2* (chr9)	0.511^a^	<0.01					Down
		circRNA *HNRNPH1* (chr11)	0.558^a^	<0.01					Down
QD Dai (2021)^13^	24 h after occlusion	circRNA *HECTD1* (brain) (chr12) (serum)	3.673^a^	≤0.001					Up
			4.106^a^	≤0.001					
B Yang (2021)^46^	24 h after occlusion	circRNA *TTC3* (chr16)	3.714^a^	<0.001					Up
ZD Zhang (2021)^47^	24 h after occlusion	circRNA *HECTD1* (chr12)	1.610^a^	<0.05					Up

^a^ Acquired from bar‐chart with WebPlotDigitizer.

^b^ Calculated with provided individual data.

^c^ Not found on multiple circRNA databases.

### 3.3 Diagnostic accuracy

**FIGURE 2:**
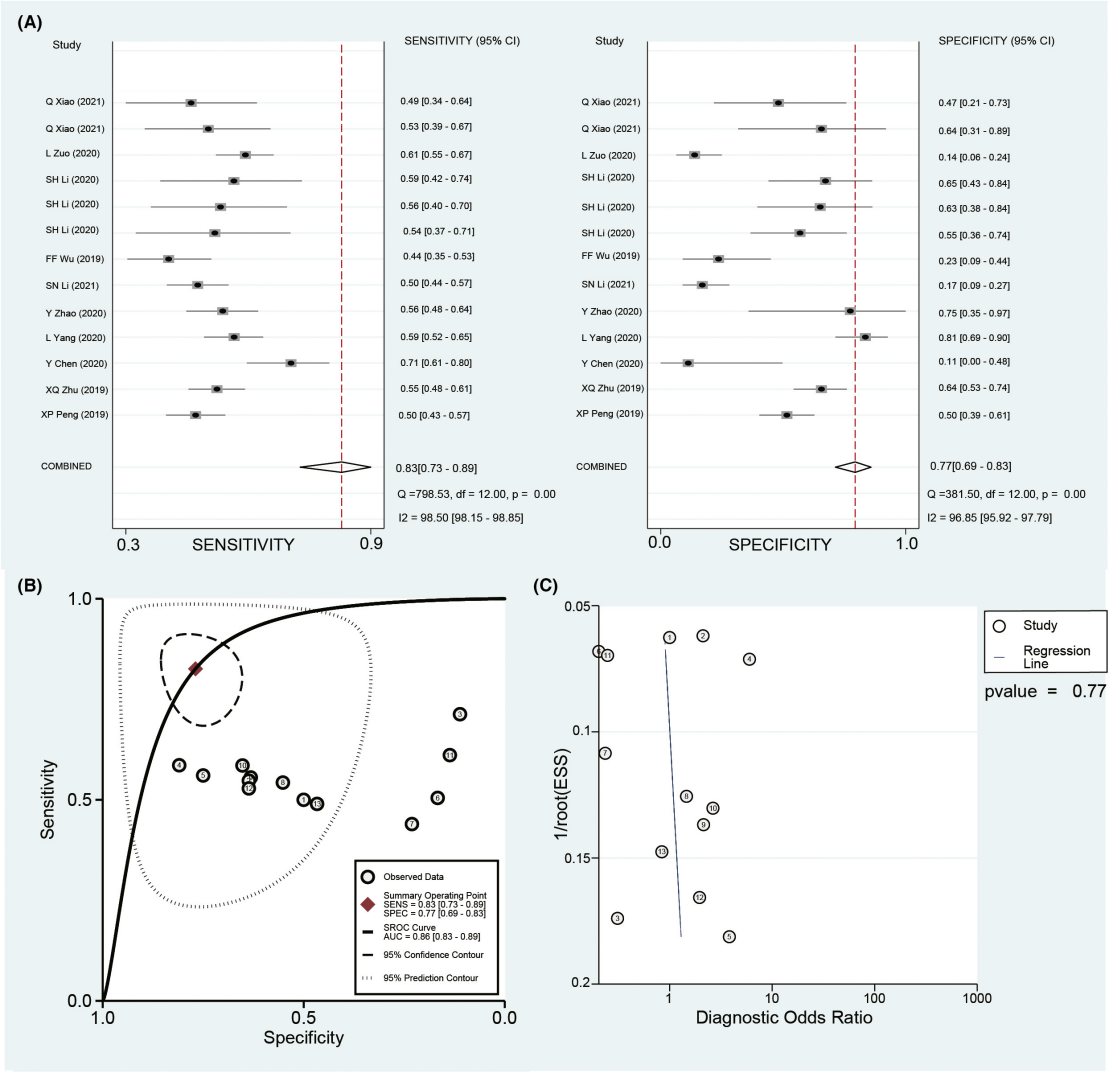
Diagnostic analysis of circular RNAs. (A) The forest plot of sensitivity and specificity of circular RNAs in the diagnosis of acute ischemic stroke (AIS). (B) Receiver operator characteristic curve of circular RNAs in diagnosing AIS. (C) The funnel plot for publication bias.

Eighteen circRNAs detected with qRT‐PCR from 10 studies involving 1148 patients and 948 controls were evaluated for their ability as biomarkers for AIS. Two studies (QX et al., 2021 and FFW et al., 2019) provided the combined diagnostic value of several circRNAs, and there were no overlapping circRNAs of concerns in these studies. Heterogeneity was assessed with *Q*‐test and *I* ^2^ statistics. Results indicated significant heterogeneity in the pooled sensitivity (*I* ^2^ = 98.50%, *p* < 0.001) and specificity (*I* ^2^ = 96.85%, *p* < 0.001), and therefore, a random‐effects model was applied. Diagnostic meta‐analysis showed the pooled sensitivity was 0.83 (95% CI, 0.73–0.89) and the pooled specificity was 0.77 (95% CI, 0.69–0.83) (Figure 2A). The pooled diagnostic odds ratio (DOR) was 18 (95% CI, 9–38) and the AUC was 0.86 (95% CI, 0.83–0.89; Figure 2B). No publication bias was detected according to the Deek's funnel plot (*p* = 0.77; Figure 2C).

### 3.4 Sensitivity analysis and meta‐regression

Because of the overall high heterogeneity in results with the random effects model, we further performed sensitivity analysis. We postulated that sample size was the main bias affecting the results, however, the results of meta‐analysis excluding two studies with the smallest sample size was still high in heterogeneity (sensitivity: *I* ^2^ = 99.37%, *p* < 0.001; specificity: *I* ^2^ = 98.46%, *p* < 0.001). Furthermore, meta‐regression suggested that standard test, index test, and subject descriptions all had no significant contribution to heterogeneity.

### 3.5 Bioinformatics analysis

**FIGURE 3:**
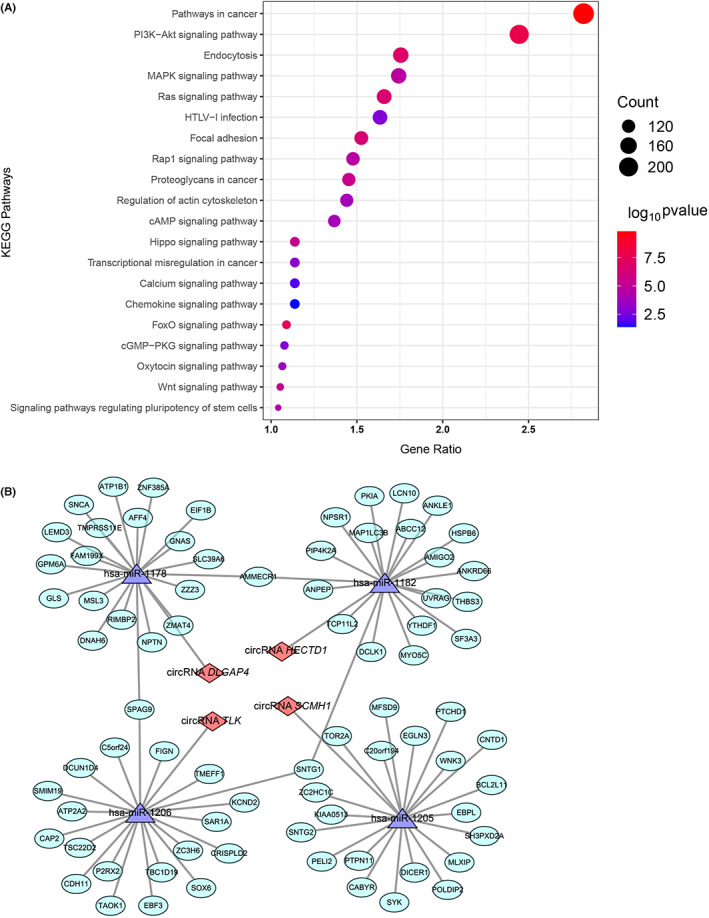
Bioinformatic analysis for the included circular RNAs. (A) The bubble plot of the top 20 enriched KEGG pathways for target genes of circRNA *HECTD1*, circRNA *DLGAP4*, circRNA *CDC14A*, circRNA *SCMH1*, and circRNA *TLK1*. (B) The ceRNA network of the top miRNA and top genes for circRNA *HECTD1*, circRNA *DLGAP4*, circRNA *SCMH1*, and circRNA *TLK1*.

CircRNAs that were reported to have the same trend of regulation in more than one human study or at least one human and one animal study were selected, which were circRNA *HECTD1*, circRNA *DLGAP4*, circRNA *TLK1*, circRNA *SCMH1*, and circRNA *CDC14A*. KEGG pathway analysis for the downstream genes of these five circRNAs and ceRNA network of the first four were shown in Figure 3A,B respectively. GO analysis described the targeted genes from three dimensions, molecular function, cellular component, and biological process. Results indicated that the targeted genes of these circRNAs mainly functioned through gene expression regulation. KEGG pathway analysis identified signaling pathways which possibly played important roles in the pathophysiology of AIS. The pathway analysis showed that most of the targeted genes functioned in metabolic pathways and pathways in cancer. As for KEGG disease analysis, they enriched in various neurological diseases, including encephalopathies, hereditary intellectual developmental disorders, etc.

## 4 DISCUSSION

Acute ischemic stroke is the second leading cause of death and disability worldwide, and timely removal of blockade was crucial for improving patients' outcomes. Therefore, it is imperative to diagnose AIS early, which currently depends largely on physicians' experience, and some accurate and practical biomarkers are expected. The circRNA is a class of noncoding RNAs and recently has been proved by many studies to be multifunctional in many diseases. Research found that brain has the most tissue‐specific circRNAs, indicating their roles in regulating bioprocesses of the brain.^11^, ^12^ Several meta‐analyses have shown that circRNAs are promising as diagnostic biomarkers and treatment targets in cardiovascular diseases and cancers,^7^, ^8^, ^9^ and some individual studies have already explored their roles in AIS. Therefore, we hoped to update the comprehensive knowledge of circRNA profile in AIS through this systemic review and meta‐analysis, which to the best of our knowledge, was the first to integrate circRNA studies in AIS patients and animal models. Results showed that although hundreds of circRNAs were reported differentially expressed in AIS patients/animals with microarray or sequencing, there lacked consistency across individual studies and different detecting technologies. Since qRT‐PCR provided accurate FC, only circRNAs tested or verified with PCR were further collected and analyzed. Furthermore, some studies explored the diagnostic value of the circRNAs. The pooled sensitivity, specificity, and DOR tests showed that circRNAs in general performed well as diagnostic biomarkers. CircRNA *HECTD1*, circRNA *DLGAP4*, circRNA *CDC14A*, circRNA *SCMH1*, and circRNA *TLK1* were reported by more than one studies to be significantly differentially expressed. Bioinformatic analysis of these five circRNAs indicated that the targeted genes were enriched in regulating cell proliferation, apoptosis, and oxidative stress.

Many experiments have explored the functions of the circRNAs reported in this study, among which circRNA *HECTD1* and circRNA *DLGAP4* were the mostly researched in relation to ischemic stroke. CircRNA *HECTD1* is derived from exons 23 and 24 of the HECT domain E3 ubiquitin protein ligase 1, *HECTD1* gene. Comparison between AIS patients and healthy controls indicated that higher circRNA *HECTD1* level was associated with higher risk disease risk and severity. Furthermore, knockdown of circRNA *HECTD1* decreased infarct areas in mice with transient middle cerebral artery occlusion (tMAO) and reduced oxygen–glucose deprivation (OGD)‐induced cell death in vitro. Several targets of circRNA *HETCD1* were proposed, including miRNA‐27a‐3p/*FSTL1*, miRNA‐133b/*TRAF3*, and miRNA‐142/*TIPARP*, and the proteins functioned in neural apoptosis and inflammation.^13^, ^14^, ^15^ While circRNA *DLGAP4*, originated from exons 8, 9 and 10 of fourth DLGAP (*DLGAP4*) gene, negatively associated with ischemic stroke development and severity, probably through influencing blood–brain barrier integrity or inflammation process. CircRNA *DLGAP4* was also reported to have protective effects in diabetic kidney disease progression, myocardial ischemia–reperfusion injury, and Parkinson's disease, all by regulating the most probable target, miR‐143, but different downstream target genes.^16^, ^17^, ^18^ We did KEGG analysis for miR‐143 alone and found that MAPK is the most enriched pathway, which is a well‐known pathway involved in proinflammation, apoptosis, and growth. Two recent human studies proved that circRNA *CDC14A*, which is made by reverse splicing of exons 5, 6 and 7 of cell division cycle (*CDC14A*) gene, was upregulated in AIS patients.^19^, ^20^ One team further researched into circRNA *CDC14A*, showing that knockdown of circRNA *CDC14A* had high expression in neurons and could ameliorate infarct sizes in tMAO mice by reducing astrocyte activation. CircRNA *SCMH1*, derived from Scm polycomb group protein homolog 1 (*SCMH1*) gene and circRNA *TLK1*, derived from exons 2 and 3 of tousled‐like kinase 1 (*TLK1*) gene, both were reported to upregulated in one human and one animal studies.^20^, ^21^ Yang L's team proved that, unlike ceRNA mechanism, circRNA *SCMH1* bound to methyl‐CpG‐binding protein 2 (MeCP 2) to upregulate downstream genes, promoting neuronal plasticity and alleviating ischemic stroke. Other studies about circRNA *SCMH1* were mainly in cancers, results of which showed that this circRNA was upregulated in cancer cells and might have the ability to promote cell proliferation in various cancer cells.^22^, ^23^, ^24^ While its function in neural cells remains to be explored. Previous studies reported that circRNA *TLK1* exacerbated myocardial ischemia–reperfusion injury and acute kidney injury by regulating inflammatory and oxidative stress, and promoted cancer progression.^25^, ^26^, ^27^ Wu FF and his colleagues found that the level of circRNA *TLK1* was increased in the blood of AIS patients and also the brain tissues of tMAO mice. Furthermore, the level of circRNA *TLK1* was more significantly increased in the large artery and small artery occlusion subtypes of stroke than in the cardinal embolism subtype. The possible target of this circRNA was TCDD inducible poly (ADP‐Ribose) polymerase (TIPARP), a transcription regulator, but the mechanisms of this protein in AIS were unclear.

KEGG pathway analysis showed the target genes mainly enriched in Phosphatidylinositol 3 kinases‐Akt (PI3K‐Akt) signaling and mitogen‐activated protein kinase (MAPK) signaling pathways. Previous studies revealed that activation of the PI3K/Akt signaling pathway could inhibit cerebral cell apoptosis, attenuating ischemia/reperfusion (I/R) injury.^28^, ^29^ MAPK signaling pathway regulates various basic cellular processes such as cellular proliferation, differentiation, migration, metabolism, and apoptosis. Mounting evidence indicated that activation of p38 MAPK and ERK1/2 MAPK was associated with inflammatory reaction, and abnormal blood–brain barrier.^30^, ^31^

Several included studies explored the feasibility of circRNA as diagnostic biomarkers for acute ischemic stroke. Peng et al.^15^ showed that the AUC (95% CI) of circRNA *HECTD1* was 0.814 (0.768, 0.859), with sensitivity and specificity of 0.725, while Zhu et al.^32^ indicated that the AUC (95% CI) of circRNA *DLGAP4* was 0.816 (0.772, 0.859), with sensitivity of 0.812 and specificity of 0.671. Lei Zuo's team and Qi Xiao's team analyzed the diagnostic value of several circRNAs combined.^19^, ^33^ We did diagnostic meta‐analysis of some available circRNAs reported in human, indicating the general promising performance of circRNAs as biomarkers for AIS.

This study has the following limitations. Because of the small number of studies exploring circRNAs in AIS and various nomenclature methods, we uniformly utilized the originating gene symbols to represent different circRNAs. Although there were previous studies that adopted this method, deviations might exist among circRNAs of different IDs. Besides, because the raw data of some studies were not public and the primary results of RNA sequencing could be complicated and probably not robust, we only did further diagnostic analysis and bioinformatic analysis of differentially expressed circRNAs detected by qRT‐PCR. In addition, there is high heterogeneity regarding the outcomes of included studies, therefore pooling these data had a risk due to inherent uncertainty. Finally, the functions of circRNAs were still relatively unclear and there is still no practical use of circRNA in clinic, more laboratory experiments were needed to validate the current results.

## 5 CONCLUSION

In conclusion, this systemic review and meta‐analysis summarizes circRNA profiling studies of acute ischemic stroke. Results illustrated the general promising performance of circRNAs as biomarkers for ischemic stroke. Five most likely important circRNAs, which were circRNA *HECTD1*, circRNA *DLGAP4*, circRNA *CDC14A*, circRNA *SCMH1*, and circRNA *TLK1*, could be the primary targets for future research.

## AUTHOR CONTRIBUTIONS

YM, LJ, and TW developed the initial idea for this study. XZ, MW, and XM developed and revised the search strategy. XZ, MW, and XM contributed to the original draft. XZ, MW, XM, GC, YL, ZH, WL, RX, JL, WL, YY, TW, LJ, and YM were responsible for the revision of the draft. XZ, MW, and XM contributed equally and are co‐first authors. All authors approved the final version of the manuscript before submission.

## FUNDING INFORMATION

The study was supported by the Natural Science Foundation of China (No. 82171303), the Beijing Scientific and Technologic Project (Z201100005520019), and Beijing Hospitals Authority's Ascent Plan (DFL20220702).

## CONFLICT OF INTEREST STATEMENT

No of the authors have any disclosures.

## Supporting information


Appendix S1

## Data Availability

The data that support the findings of this study are available from the corresponding author upon reasonable request.
