# Supplementary material for: Circular RNA as biomarkers for acute ischemic stroke: A systematic review and meta‐analysis
Source: CNS Neurosci Ther. 2023 Apr 26;29(8):2086–100. doi: 10.1111/cns.14220 (PMC10352895; doi:10.1111/cns.14220)
Supplement: Supplementary file 1 — Appendix S1 [file CNS-29-2086-s001.docx]

| Study | Course design | Description of the samples | Representation of case and control | Annotation of platform and naming of circRNAs | Data processing and analysis |
| --- | --- | --- | --- | --- | --- |
| XP Peng (2019) | 2 | 2 | 2 | 1 | 2 |
| XQ Zhu (2019) | 2 | 1 | 2 | 1 | 1 |
| Y Chen (2020) | 2 | 1 | 1 | 1 | 2 |
| ZF Dong (2020) | 2 | 2 | 2 | 2 | 1 |
| SH Li (2020) | 2 | 0 | 1 | 2 | 2 |
| L Yang (2020) | 2 | 2 | 2 | 1 | 1 |
| Y Zhao (2020) | 2 | 1 | 1 | 1 | 1 |
| L Zuo (2020) | 2 | 1 | 1 | 0 | 2 |
| SN Li (2021) | 2 | 2 | 1 | 1 | 2 |
| Q Xiao (2021) | 2 | 1 | 0 | 1 | 2 |
| Y Bai (2018) | 2 | 2 | 2 | 1 | 1 |
| B Han (2018) | 2 | 2 | 2 | 1 | 1 |
| FF Wu (2019) | 2 | 2 | 1 | 2 | 2 |
| D Lu (2020) | 2 | 1 | 1 | 2 | 2 |
| S. L. Mehta (2017) | 2 | 1 | 2 | 2 | 1 |
| XL Yang (2018) | 2 | 0 | 2 | 1 | 1 |
| WH Chen (2020) | 2 | 0 | 2 | 1 | 1 |
| CG Tang (2020) | 2 | 0 | 2 | 1 | 1 |
| LQ Wu (2020) | 2 | 1 | 2 | 1 | 1 |
| ZH Zhang (2020) | 2 | 1 | 2 | 2 | 1 |
| QD Dai (2021) | 2 | 1 | 2 | 1 | 1 |
| B Yang (2021) | 2 | 1 | 2 | 1 | 1 |
| ZD Zhang (2021) | 2 | 1 | 2 | 1 | 1 |

**Table S1. Quality assessment of the included studies**

**Table S2. Quality Assessment of Diagnostic Accuracy Studies 2 (QUADAS-2) of the included studies**

| Study | Patient selection | Index test | Reference standard | Flow and timing |
| --- | --- | --- | --- | --- |
| XP Peng (2019) | Unclear | Unclear | High | High |
| XQ Zhu (2019) | High | Unclear | Unclear | High |
| Y Chen (2020) | Unclear | Unclear | Unclear | Low |
| SH Li (2020) | Unclear | Unclear | Unclear | Unclear |
| L Yang (2020) | High | Unclear | High | Low |
| Y Zhao (2020) | Low | Unclear | High | High |
| L Zuo (2020) | Low | Unclear | High | Low |
| SN Li (2021) | Low | Unclear | High | High |
| Q Xiao (2021) | Low | Unclear | Low | Low |
| FF Wu (2019) | Unclear | Unclear | Unclear | High |
